# Proteomic and Metabolomic Profiling Reveals Alterations in Boar X and Y Sperm

**DOI:** 10.3390/ani14243672

**Published:** 2024-12-19

**Authors:** Jia Cheng, Xu Hao, Weijing Zhang, Chenhao Sun, Xiameng Yuan, Yiding Yang, Wenxian Zeng, Zhendong Zhu

**Affiliations:** 1 School of Biological Science and Engineering, Shaanxi University of Technology, Hanzhong 723001, China; chengjia@snut.edu.cn (J.C.); hx694410665@163.com (X.H.); sch1225bx@163.com (C.S.); 19999752282@163.com (X.Y.); y13609295038@163.com (Y.Y.); 2 Qinba Mountain Area Collaborative Innovation Center of Bio-Resources Comprehensive Development, Hanzhong 723001, China; 3 Qinba State Key Laboratory of Biological Resources and Ecological Environment (Incubation), Hanzhong 723001, China; 4 College of Animal Science and Technology, Qingdao Agricultural University, Qingdao 266109, China; zhangweijing202103@163.com

**Keywords:** boar, sex-controlled sperm, proteomics, metabolomics

## Abstract

Sex-controlled sperm combined with artificial insemination allows animals to reproduce offspring according to the desired sex, accelerates the process of animal genetics and breeding and promotes the development of animal husbandry. However, the molecular markers for sexual sperm sorting are unusual. To identify the molecular markers of boar sperm sorting, proteomics and metabolomics techniques were applied to analyze the differences in proteins and metabolism between X and Y sperm. Label-free quantitative proteomics identified 254 differentially expressed proteins (DEPs) in the X and Y sperm of boars, including 106 proteins that were highly expressed in X sperm and 148 proteins that were highly expressed in Y sperm. Among the differential proteins, COX6A1, COX1, CYTB, FUT8, GSTK1 and PFK1 were selected as potential biological markers for X and Y sperm sorting. Moreover, 760 metabolites from X and Y sperm were detected. There were 439 positive ion mode metabolites and 321 negative ion mode metabolites identified. The various metabolites were phosphoenolpyruvate, phytosphingosine, L-arginine, N-acetylputrescine, cytidine-5′-diphosphate and deoxyuridine. These metabolites were mainly involved in the TCA cycle, oxidative phosphorylation pathway, glycolysis pathway, lipid metabolism pathway, amino acid metabolism pathway, pentose phosphate pathway and nucleic acid metabolism pathway. The differential proteins and differential metabolites obtained by the combined proteomics and metabolomics analysis were projected simultaneously to the KEGG pathway, and a total of five pathways were enriched, namely oxidative phosphorylation pathway, purine metabolism, unsaturated fatty acid biosynthesis, ABC transporters and peroxisomes. In summary, COX6A1 and CYTB were identified as potential biomarkers for boar X and Y sperm sorting.

## Simple Summary

Decoding the specific traits of X and Y sperm in animal husbandry is essential for accelerating breeding applications. However, there are numerous challenges in distinguishing between X and Y sperm. Here, we investigate the total differential expression proteins and metabolites of the two sperm types using label-free quantitative proteomics and non-targeted metabolomics analysis. The results suggest that the differential proteins and metabolites mainly enrich the energy metabolism processes in boar X and Y sperm. COX6A1 and CYTB are identified as potential biomarkers for boar X and Y sperm sorting. Our findings contribute to the methods of applying sex-sorted boar semen.

## 1. Introduction

In the field of artificial insemination, the technology of X/Y sperm sorting has significant implications for meeting the need for sex-specific offspring in animal husbandry. However, there were numerous challenges in distinguishing between X and Y sperm. Early studies focused on morphological and kinematic differences between X and Y sperm, with observations suggesting that X sperm typically have larger heads than Y sperm [1]. However, subsequent studies using more precise atomic force microscopy (AFM) failed to detect significant morphological or size differences between X and Y sperm [2]. Kinematics, observations using albumin gradient methods, suggested that Y sperm accumulated faster than X sperm at the bottom of the gradient, indicating potentially higher motility of Y sperm [3]. Nevertheless, several studies using flow cytometry and computer-assisted sperm analysis (CASA) techniques found insignificant differences in speed or swimming patterns between X and Y sperm [4]. These discrepancies are primarily attributed to the insufficient precision of previous research methods in distinguishing between X and Y sperm.

Sperm sex sorting technologies primarily encompass flow cytometry and magnetic-activated cell sorting (MACS). Flow cytometry exploits the differential DNA content between X and Y chromosome-bearing spermatozoa, with X-bearing sperm containing approximately 3.8–4.2% more DNA than Y-bearing sperm [5]. In commercial applications, this technology has demonstrated exceptional efficacy in bovine sperm sex sorting, achieving sorting purities ranging from 85% to 95% [6]. Magnetic-activated cell sorting utilizes the conjugation of specific antibodies with magnetic nanoparticles to facilitate the enrichment and separation of target spermatozoa. Studies have demonstrated that through the implementation of single-chain variable fragment (scFv) antibody labeling technology, this method can achieve X-bearing sperm purities of 82.65%, while Y-bearing sperm elution samples exhibit purities of 81.43% [7].

However, both technologies exhibit limitations in sperm sex sorting applications. Flow cytometric sorting may induce chemical and mechanical damage to spermatozoa, potentially compromising their viability and fertilizing capacity [8]. The efficiency of magnetic-activated cell sorting depends on the specific binding between antibodies and target sperm surface antigens, which can reduce the uniformity of sorting outcomes [7]. Consequently, there is a need for novel sorting technologies to enhance efficiency and sperm survival rates while reducing costs.

In recent years, omics research has made notable progress in X/Y sperm sorting, particularly in the field of proteomics. Studies have screened for differentially expressed proteins in various mammalian species, including cattle and swine. These proteins may play crucial roles in sperm motility, acrosome reaction and fertilization processes, showing potential as promising biological markers for X/Y sperm separation [9,10,11]. In contrast, metabolomics research in X/Y sperm sorting remains relatively limited, although it has demonstrated promising applications in other areas of sperm research [12,13].

In summary, omics approaches have shown great potential in identifying differential markers between X and Y sperm. The aim of this study is to perform a combined proteomic and metabolomic analysis of porcine X/Y sperm to identify additional potential biomarkers, thereby supporting future developments in sperm sorting technologies.

## 2. Materials and Methods

### 2.1. Samples Collection from Adult Boars

The sperm samples were obtained from six healthy adult Landrace boars, which were gifted by a breeding boar station from Shandong Province, China. The samples were sorted into X and Y sperm by flow cytometry. The purities of X and Y sperm after separation, as determined by quantitative polymerase chain reaction (qPCR), were more than 92% and 95%, respectively.

### 2.2. Total Protein Extraction and Digestion

The sperm samples were centrifuged at room temperature and 2000 r/min in a 15 mL centrifuge tube for 3 min. The cells were then washed twice with cold PBS and centrifuged at 4 °C and 2000 r/min for 3 min. The RIPA lysate containing the proteinase inhibitor was added to the sample sediment. The ultrasonic processor was used to completely lyse sperm in the ice condition. The lysate was centrifuged at 4 °C and 12,000 r/min for 10 min, and the supernatant was collected in a new tube for protein extraction. Finally, the protein concentration was determined by the BCA assay (E112, Vazyme, Nanjing, China).

A total of 30 μg of protein per sample was added with 1 mol/L DTT solution and boiled in water for 5 min, and then the tubes were cooled to room temperature. Subsequently, the sample was mixed well with 200 μL UA buffer [8 mol/L Urea, 150 mmol/L Tris-HCl, pH = 8.0]. Then, it was transferred to an ultrafiltration centrifuge tube and centrifuged at 12,000 r/min for 15 min to remove the waste liquid. A total of 100 μL of IAA [50 mmol/L IAA solution in UA buffer] was mixed and incubated for 30 min at room temperature in the dark. The liquid was removed after centrifugation at 12,000 r/min for 10 min. Next, the UA buffer was added to the tube and centrifuged, which was repeated twice. The precipitate was cleaned with 100 μL of NH_4_HCO_3_ buffer and collected by centrifugation at 14,000 r/min for 10 min twice. Subsequently, 40 μL of trypsin buffer was applied to incubate the samples overnight at 37 °C. The lipid was then collected in a new tube after centrifugation. Finally, 5–10 μL of 0.1% TFA was added to the filtrate, and the enzymatically digested peptides were desalted using a C18 cartridge and lyophilized under vacuum. The peptides were dried and re-dissolved with 0.1% TFA, and the peptide concentration was determined for LC-MS/MS analysis [14].

### 2.3. Protein Quantification and Bioinformatics Analysis of DEPs

The peptides were searched and identified using the Proteome Discoverer 2.4 software. Gene Ontology (GO) enrichment analysis and Kyoto Encyclopedia of Genes and Genomes (KEGG) pathway enrichment analysis were performed to annotate all identified proteins. In addition, the subcellular localization of the proteins of X/Y sperm was analyzed. All differential expression proteins were screened to identify whether a satisfactory *p* < 0.05 and fold change of > 2 or <1/2 occurred, and the differential proteins were subjected to functional enrichment and cluster analysis. Above, the data were visualized simultaneously.

### 2.4. Western Blotting

Total proteins were electrophoresed in 10% SDS-PAGE gels and transferred to PVDF membranes. After blocking for 1 h at room temperature in TBST (0.05% Tween-20 in 20 mol/L Tris-HCl, 140 mol/L NaCl, pH 7.5) with 5% BSA (ST2249, Beyotime, Shanghai, China), the PVDF was incubated overnight at 4 °C with the rabbit polyclonal antibody COX6A1 (11460-1-AP, Proteintech, Wuhan, China) and the rabbit polyclonal antibody MT-CYB (A9762, ABclonal, Wuhan, China), respectively. α-Tubulin (AC007, ABclonal, China) was used as an internal reference. After washing with TBST three times, the PVDF was incubated with horseradish peroxidase (HRP)-conjugated goat anti-rabbit IgG (A0208, Beyotime, China) for 1 h at room temperature. After washing with TBST three times, the PVDF was detected using ECL reagent (E411, Vazyme, China). Three replicates were performed as validation samples in boar X/Y sperm.

### 2.5. Non-Targeted Metabolomics Analysis and Identification of DEMs

All samples were separated on a SHIMADZU-LC30 ultra-high performance liquid chromatography (UHPLC) system using a HILIC column. The injection volume was 5 µL, the column temperature was 25 °C and the flow rate was 0.3 mL/min. The mobile phase A was H_2_O + 25 mM ammonium acetate, and the mobile phase B was acetonitrile. The gradient elution procedure was as follows: 0–1 min, 95% B; 1–7 min, B varied linearly from 95% to 65%; 7–9 min, B varied linearly from 65% to 35%; 9–10 min, B was maintained at 35%; 10–11 min, B changed linearly from 35% to 95%; and 11–15 min, B was maintained at 95%. Throughout the analysis, samples were placed in an autosampler at a temperature of 4 °C. To monitor and evaluate the stability of the system and the reliability of the experimental data, QC samples were inserted into the sample queue.

Each sample was detected by electrospray ionization (ESI) in positive (+) and negative (−) modes, respectively. Samples were separated by UPLC and analyzed by mass spectrometry on a QE Plus mass spectrometer (Thermo Scientific). Ionization was performed using a HESI source with the following ionization conditions: spray voltage, 3.8 kV (+) and 3.2 kV (-); capillary temperature, 320 (±); sheath gas, 30 (±); aux gas, 5 (±); probe heating temperature, 350 (±); and S-Lens RF level, 50.

The MS acquisition settings were as follows: MS acquisition time, 12 min; parent ion scan range, 80–1200 *m*/*z*; primary MS resolution, 70,000 @ *m*/*z* 200; AGC target, 3 × 10^6^; and primary Maximum IT, 100 ms. The secondary MS analysis was acquired in the following way, and the acquisition was triggered after each full scan. Secondary mass spectrometry (MS2 scan) of the 10 highest intensity parent ions: secondary mass resolution, 17,500 @ *m*/*z* 200; AGC target, 1 × 10^5^; secondary Maximum IT, 50 ms; MS2 Activation Type, HCD; Isolation window, 2 *m*/*z*; normalized collision energy (SSC), 1 × 10^6^; AGC target, 3 × 10^6^; and primary Maximum IT, 100 ms. Secondary mass analysis was acquired as follows: triggered after each full scan (SSC). Normalized collision energy (Setpped) was 27, 29 and 32.

Raw data were format converted and MSDIAL software (v4.60) was used for peak alignment, retention time correction and peak area extraction. Metabolite structure identification was performed by exact mass number matching (<25 ppm) and secondary spectral matching. The extracted data were deleted for ion peaks with >50% missing values within the group, positive and negative ion peaks were integrated and pattern recognition was performed using SIMCA-P 14.1 (Umetrics, Umea, Sweden) software, and the data were pre-processed by UV (Unit Variance Scaling) for multidimensional statistical analysis. Fold change > 1.5 and *p* < 0.05 were set as the thresholds for significantly differentially expressed metabolites (DEMs).

### 2.6. Integration Analysis of DEPs and DEMs

The results of the KEGG analysis were obtained through a comprehensive comparison of label-free quantitative proteomics and non-target metabolomics data. The KEGG comparison results were visualized using TBtools and the online website Figdraw (https://www.figdraw.com/, accessed on 20 January 2022). Subsequently, the KEGG pathway was correlated through comparative analysis and enrichment analysis.

### 2.7. Statistical Analysis

All data were initially sorted in Excel, and data from the fermentation experiment were analyzed using one-way ANOVA. Subsequent data were analyzed using SPSS (version 20.0), and differences between X/Y sperm groups were analyzed using the *t*-test. The results are expressed as mean ± standard deviation (SD), and *p* < 0.05 was considered to indicate a statistically significant difference.

## 3. Results

### 3.1. Proteomic Profiling Features Analysis of Boar X/Y Sperm

**Figure 1:**
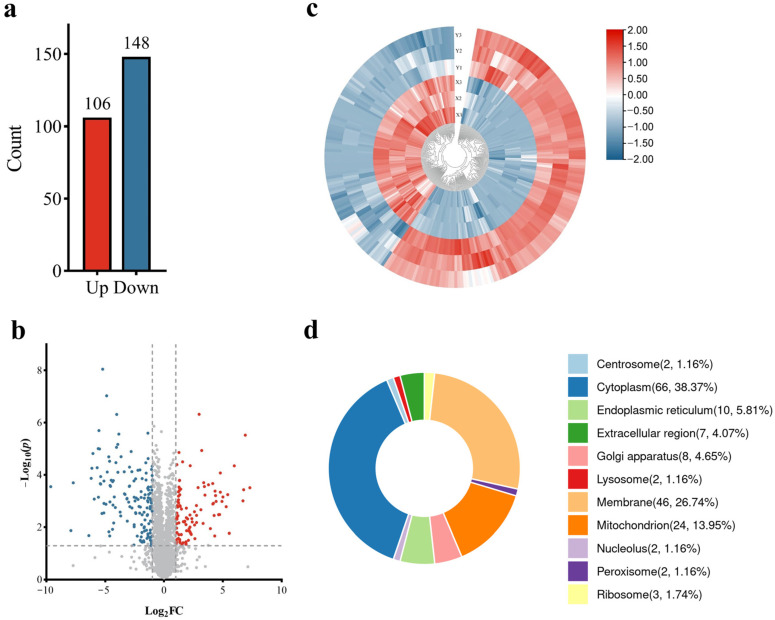
(**a**) The counts of differentially expressed proteins (DEPs) in comparison in boar X/Y sperm (X sperm vs. Y sperm). (**b**) Volcano plot displaying significant DEPs between two different comparison groups. (**c**) Heatmap of significant DEPs within boar X/Y sperm. (**d**) Subcellular localization of significant DEPs within two different comparison groups.

The boar X/Y sperm samples were used in a proteomic study. The quantitative proteomic analysis of sex-specific antigens was performed using a label-free quantitative method. The results of this protein mass spectrometry data analysis showed that the total number of identified secondary profiles was 284,515, the total number of identified peptides was 21,438, the number of combined proteins was 2399 and the total number of proteins with quantitative results was 2272 (Figures S1 and S2). According to the screening criteria of fold change > 2 or <0.5 and *p*-value ≤ 0.05, a total of 254 significantly different expression proteins (DEPs) of X and Y sperm were screened, of which 106 were up-regulated and 148 were down-regulated (Figure 1a,b). The identified top 10 DEPs in the X sperm group and the Y sperm group were listed, respectively (Table 1 and Table 2).

Clusters of all significantly differentially expressed proteins were visualized by a heatmap (Figure 1c), which suggested that the data were well clustered between or within groups. Meanwhile, the differential proteins were found to be mainly localized in the cytoplasm, membrane, mitochondria and endoplasmic reticulum (Figure 1d). Compared with other organelles, the proportion of DEPs in cytoplasm and membrane was 65.11%, showing that both were significant in boar X/Y sperm.

### 3.2. Biological Differences Between Boar X and Y Sperm

**Figure 2:**
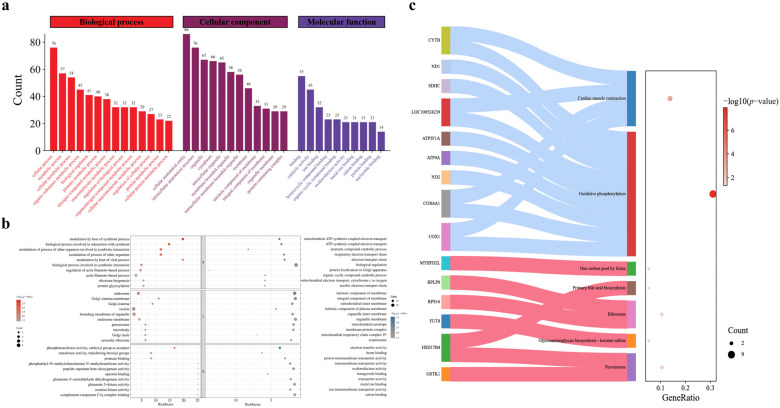
(**a**) GO annotations map of DEPs in boar X/Y sperm. The histogram represents the number of genes contained in the biological process, cellular component and molecular function, respectively. (**b**) Bubble diagram of the GO function with significant DEPs in boar X/Y sperm, *p* value < 0.05. The size of the bubbles represents the number of genes involved in the pathway. (**c**) KEGG enrichment analysis of up/down-regulated DEPs in boar X/Y sperm.

To assess the biological significances of the DEPs in boar X/Y sperm, the proteins were further classified based on a series of bioinformatics analyses. For the “biological process” aspect, GO terms were mainly associated with the cellular processes, metabolic processes, cellular metabolic processes, organic metabolic processes, and biological regulation. For the “cellular component”, the DEPs were associated with cellular anatomical entities, internal cellular anatomical units, organelles, and cytoplasm. For “molecular function”, classification analysis revealed that most DEPs were associated with gene binding activity, catalytic activity, ion binding, and oxidoreductase activity (Figure 2a).

The GO functional enrichment analysis of up-/down-regulated differential proteins revealed that DEPs in X sperm were mainly involved in the actin regulation process, which was related to endosomes, endosomal membrane composition and phosphotransferase activity. However, the DEPs in Y sperm were mainly involved in ATP synthesis and the electron transfer process, which was related to the intrinsic components of the cell membrane, and the electron transfer activity was stronger (Figure 2b). Meanwhile, KEGG functional enrichment analysis showed that the significantly different pathways enriched for up-regulated proteins were peroxisome and ribosome. On the other hand, the significantly different pathway enriched for down-regulated proteins was the oxidative phosphorylation pathway (Figure 2c and Tables S1 and S2).

**Figure 3:**
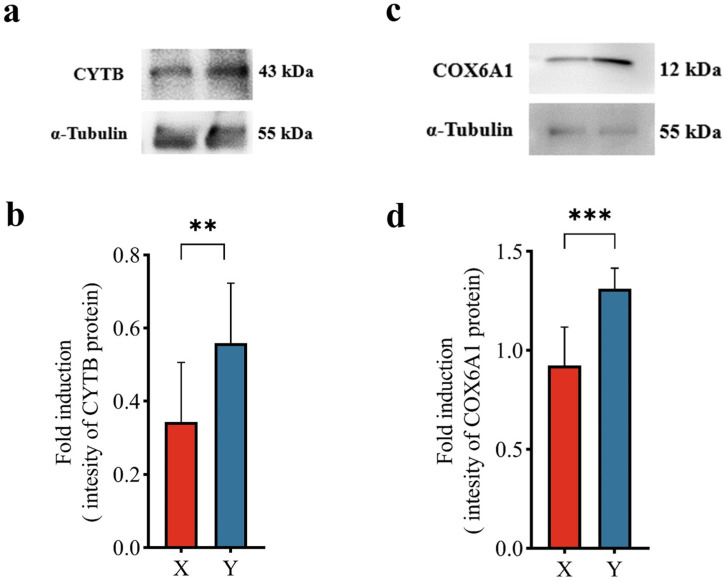
(**a**,**c**) Western blotting verification results for CYTB and COX6A1, respectively. α-tubulin was detected as housekeeping control. (**b**,**d**) Gray statistics of CYTB and COX6A1, respectively. ** *p* value < 0.01. *** *p* value < 0.001.

Among DEPs, 21 proteins were closely related to energy metabolism, with 8 proteins highly expressed in X sperm and 13 proteins highly expressed in Y sperm. FUT8 (alpha-(1,6)-fucosyltransferase), GSTK1 (glutathione S-transferase kappa), PGK1 (phosphoglycerate kinase), COX6A1 (cytochrome c oxidase subunit), COX1 (cytochrome c oxidase subunit 1) and CYTB (cytochrome b) were selected as potential markers in X/Y sperm for subsequent studies related to subcellular localization and annotation of gene function. Based on proteomic analysis, Western blotting (WB) results showed that CYTB and COX6A1 were more highly expressed in Y sperm than in X sperm (Figure 3).

### 3.3. Identification of Different Expression Metabolites in Boar X/Y Sperm

To evaluate the metabolomic changes induced by X/Y sperm, an untargeted screening of boar sperm samples was performed (Figure S3). Based on the PCA and OPLS-DA analysis, discriminated metabolites that contributed to the difference in metabolomics in pairwise comparisons were labeled with their VIP, fold change and *p*-value for both positive and negative ion modes (Table 3 and Figure S4). The results indicated the goodness of fit and the predictive ability of the model. In our study, a total of 760 metabolites were identified, with 439 and 321 metabolites identified in positive and negative ion modes, respectively.

**Figure 4:**
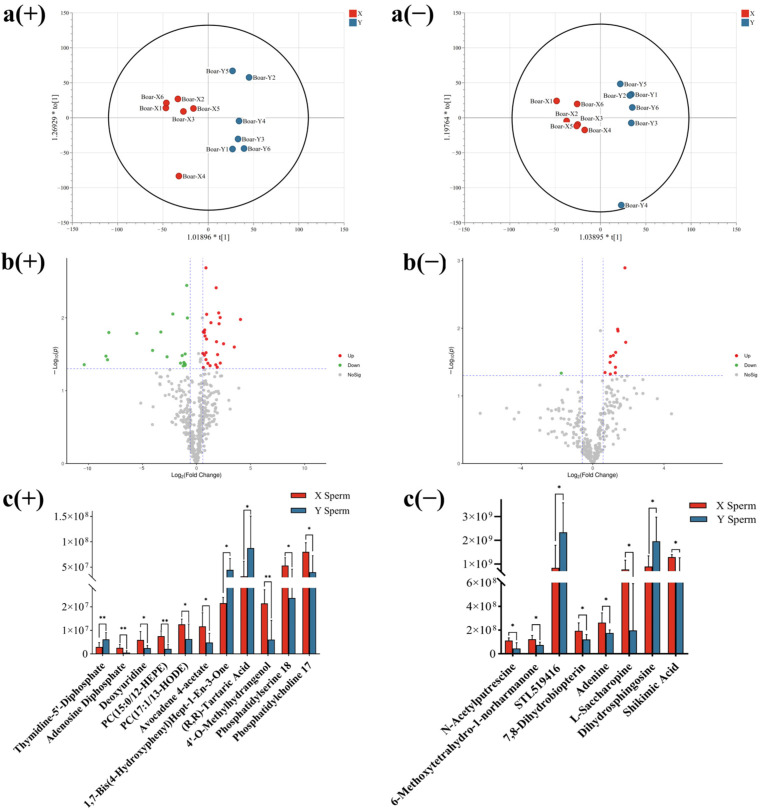
(**a**) Comparative OPLS-DA analysis of positive (+) and negative (−) ionic mode detected two sperm types. (**b**) Comparative volcano plots of positive (+) and negative (−) ionic mode detected two sperm types; fold change > 1.5 or fold change < 0.667 and *p* < 0.05. (**c**) Relative expression of DEMs of positive (+) and negative (−) ionic mode detected boar X/Y sperm, * *p* value < 0.05. ** *p* value < 0.01.

The differential expression metabolites (DEMs) between X/Y sperm groups were screened out by adjusting the statistical standards at fold change > 1.5 or fold change < 0.667 and a *p* < 0.05, which were summarized in volcano plots for both positive and negative ion modes. As a consequence, 27 DEMs were up-regulated and 17 DEMs were down-regulated in positive ion mode, while 12 DEMs were up-regulated and only 1 DEM was down-regulated in negative ion mode (Figure 4).

### 3.4. Metabolic Pathways of Differential Metabolites

**Figure 5:**
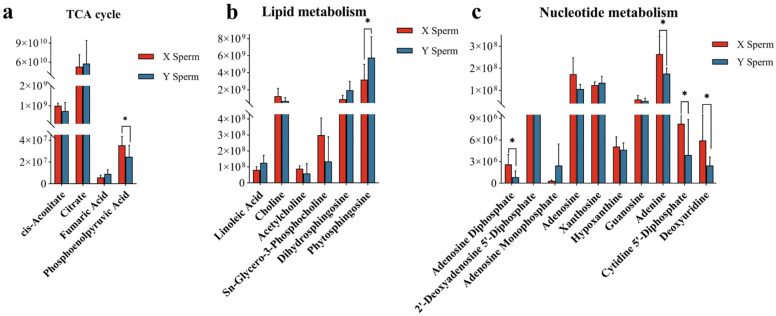
(**a**) Pairwise comparison of the discriminated metabolites changed in TCA cycle. * *p* value < 0.05. (**b**) Pairwise comparison of the discriminated metabolites changed in lipid metabolism. * *p* value < 0.05. (**c**) Pairwise comparison of the discriminated metabolites changed in nucleotide metabolism. * *p* value < 0.05.

The KEGG pathway enrichment provided an overall picture of variations in metabolite profile caused by varying protein levels. KEGG enrichment of DEMs related to the energy metabolism pathway was analyzed. The results indicated that phosphoenolpyruvate, phosphatidylinositol, L-arginine, N-acetylputrescine, cytidine 5′-diphosphate and deoxyuridine were DEMs between X/Y sperm. These DEMs were mainly involved in the tricarboxylic acid (TCA) cycle, lipid metabolism, nucleic acid metabolism and amino acid metabolic pathways (Figure 5 and Figure S5), which suggested that boar X/Y sperm had alterations in the level of the metabolites. Specifically, most DEMs were higher in X sperm than Y sperm, such as phytosphingosine in the TCA cycle; adenosine diphosphate, adenine, cytidine 5′-diphosphate and deoxyuridine in nucleotide metabolism; and L-arginine and N-acetyl putrescine in amino metabolism. Conversely, phytosphingosine levels were detectably higher in Y sperm involved in lipid metabolism.

### 3.5. Altered Pathways Enriched by DEMs and DEPs

**Figure 6:**
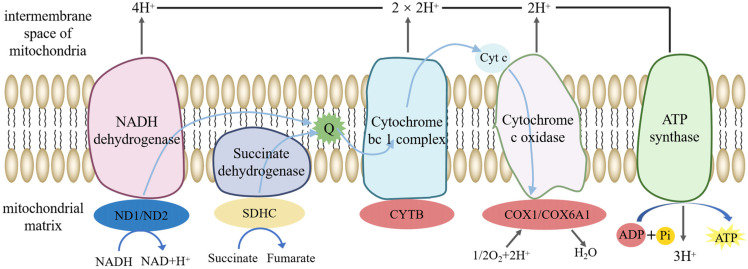
Oxidative phosphorylation pathway. Note: The oval in the KEGG figure represents the protein, the circle represents the metabolite; the red/blue indicates up-/down-regulated proteins/metabolites; and yellow indicates that proteins/metabolites detected but that there were no differences.

By mapping DEPs and DEMs associated with energy metabolism to KEGG, pathways such as oxidative phosphorylation, purine metabolism, unsaturated fatty acids synthesis, ABC transporters and peroxisomes were enriched. The DEPs and DEMs associated with oxidative phosphorylation are shown in Figure 6. Since sperm motility is positively correlated with its metabolic level, both DEPs and DEMs determined the boar’s X/Y sperm motility performance.

## 4. Discussion

In animal husbandry, sex control technology optimizes sex-limited traits and maximizes economic benefits, enhances selection intensity, and accelerates breeding progress. Additionally, it helps to address issues such as freemartins and sex-linked detrimental genes. Therefore, sex control technology effectively improves livestock production efficiency and accelerates breeding progress [15]. In recent years, flow cytometry has been the main technology used in the industrialization of sperm sexing. This technology offers advantages such as minimal damage to sperm and short operation times. However, a disadvantage of flow cytometry is the relatively low efficiency in producing sufficiently sorted sperm for artificial insemination [16], which represents a limitation to the widespread use of a flow sorter in the pig industry [17].

In parallel, the development of new technologies and strategies contributes to the application of sex control in livestock production. Omics technologies are used to efficiently screen for differences between X and Y sperm in many pieces of research. The specific antigens CLRN3 and SCAMP1 were identified on the cell membrane of X/Y sperm in Holstein bulls based on proteomic assay [9]. In X/Y sperm from *Bos indicus* cattle, the differentially expressed proteins were mainly related to structural molecular activity, GTP/ATP binding and GTPase/ATPase activity [18]. To investigate the sex-specific candidate proteins in boar semen, the proteomic research in the study identified 254 DEPs, with 106 highly expressed in X sperm and 148 in Y sperm. A number of DEPs were involved in energy metabolism processes, in agreement with one previous study [11].

Theoretically, the membrane protein-based immunological methods could improve the accuracy of sperm sorting and ensure the selection of sperm with higher reproductive potential. While our proteomic results revealed that among the differential proteins related to energy metabolism, most Y sperm differential proteins were enriched in the oxidative phosphorylation pathway. Among DEPs in porcine Y sperm, CYTB expression was highly increased and is closely related to mitochondrial complex III function and ATP generation [19,20]. Meanwhile, this study found that COX6A1 expression increases in Y sperm, manifested by stronger Y sperm motility and better fertilization ability [21]. Both proteins were further verified by Western blot, suggesting that CYTB and COX61 could serve as potential biomarkers for X/Y sperm sorting. In addition, succinate dehydrogenase complex subunit C (SDHC) is a subunit of mitochondrial respiratory chain complex II, which can reduce O_2_^−^ production [22] and serves as a biomarker for assessing the quality of frozen goat semen [23].

Compared to Y boar sperm, energy metabolism-related proteins highly expressed in X sperm include GSTK1, phosphatidylethanolamine N-methyltransferase (PEMT) and PGK1. Several studies have shown that GSTK1 plays a crucial role in reproduction and high-fertility sperm [24,25,26,27]. PEMT transcripts have also been detected in human testes, and PEMT may be associated with high sperm concentration [28]. Its differential expression in X/Y sperm was clearly defined in the present study. PGK1 may be associated with high fertility, but the specific mechanism of action remains unclear [29]. Therefore, further screening and identifying of X and Y sperm-specific proteins or DEPs is essential. The DEPs in porcine X and Y sperm were involved in the regulation of the metabolic pathway, mainly resulting in metabolomic changes. The analysis of differential metabolites revealed that they are differential metabolites related to energy metabolism. Moreover, most of these DEMs were found at higher concentrations in X sperm, which contrasts with the proteomics results. It is worth noting that X/Y sperm adopt different energy metabolism strategies, which could be closely related to sperm function and motility. This suggests that artificial optimization of the boar semen extender could be considered to maximize the differences between live X and Y sperm [30]. Ren et al. reported that the Toll-like receptor 7/8 (TLR7/8) was localized in the midpiece and tail of X sperm, and the activation of TLR7/8 ligands selectively inhibited X sperm motility without affecting Y sperm [31]. When sperm treated with the TLR7/8 agonist (R848) were used for in vitro fertilization, 90% of the embryos were male [32]. This suggests that sex control could be achieved through the selective inhibition of Y sperm motility. At this point, further research targeting CYTB- or COX6A1-specific small molecule inhibitors could contribute to increasing the sorting efficiency. However, reduced CYTB expression could result in asthenozoospermia [20], indicating that female offspring production may increase when a CYTB inhibitor is used without affecting fertility rates. The application of sex sorting in the porcine production systems still presents several challenges.

This study revealed differences in energy metabolism between porcine X and Y sperm through proteomic and metabolomic analyses. These findings not only deepen our understanding of the physiological characteristics of X and Y sperm but also provide a theoretical basis for potential future sex control methods.

## 5. Conclusions

COX6A1 and CYTB mainly regulated the oxidative phosphorylation pathway, which is involved in alterations in the level of the metabolites in boar X/Y sperm. Both could be identified as potential biomarkers for boar X and Y sperm sorting.

## Figures and Tables

**Table 1:** Top 10 proteins with the highest expressions in X sperm.

Gene Names	Protein Names	Fold Change	*p* Value
CAPZB	F-actin-capping protein subunit beta	156.57	3.11 × 10^−4^
FTL	Ferritin light chain (Fragment)	120.24	3.02 × 10^−6^
ANXA2	Annexin	62.47	4.51 × 10^−5^
DNAJC13	J domain-containing protein	46.52	1.70 × 10^−2^
HIGD1A	HIG1 domain family member 1A	42.61	5.68 × 10^−4^
TNFAIP2	TNF alpha-induced protein 2	38.93	1.66 × 10^−3^
LGMN	Asparaginyl endopeptidase	32.14	6.97 × 10^−4^
INTS7	Integrator complex subunit 7	27.47	8.47 × 10^−5^
LOC110255254	MAGE domain-containing protein	24.81	3.33 × 10^−3^
LRRC74A	Leucine-rich repeat-containing 74A	21.55	1.04 × 10^−3^

**Table 2:** Top 10 proteins with the highest expressions in Y sperm.

Gene Names	Protein Names	Fold Change	*p* Value
COX6A1	Cytochrome c oxidase subunit	0.0041	1.36 × 10^−2^
TKFC	ATP-dependent dihydroxyacetone kinase	0.0119	2.06 × 10^−2^
CDKL3	Cyclin-dependent kinase like 3	0.0151	1.52 × 10^−5^
PRDX4	Thioredoxin domain-containing protein	0.0206	9.84 × 10^−6^
SMC3	Structural maintenance of chromosomes protein	0.0209	7.17 × 10^−4^
CCT3	T-complex protein 1 subunit gamma	0.0215	2.01 × 10^−6^
TRAPPC6B	Trafficking protein particle complex 6B	0.0222	1.02 × 10^−5^
CSNK1D	Casein kinase 1 delta	0.0238	1.16 × 10^−4^
VKORC1	Vitamin-K-epoxide reductase (warfarin-sensitive)	0.0239	2.24 × 10^−4^
ATF6	Activating transcription factor 6	0.0257	3.01 × 10^−3^

**Table 3:** Differential expression metabolites of boar X/Y sperm.

Metabolites	Fold Change	*p* Value	VIP	Ionic Mode
Adenosine Diphosphate	4.57	0.99 × 10^−2^	1.81	POS
L-Saccharopine	3.87	3.20 × 10^−2^	1.57	POS
(3R)-8-Hydroxy-3-(4-Methoxyphenyl)-3,4-Dihydroisochromen-1-One	3.53	0.39 × 10^−2^	1.95	POS
N-Acetylputrescine	2.52	1.17 × 10^−2^	1.77	POS
Shikimic Acid	1.84	3.01 × 10^−2^	1.547	POS
1,2,3,4-Tetrahydro-6-Methoxy-1-Oxo-Beta-Carboline	1.65	1.59 × 10^−2^	1.62	POS
Adenine	1.50	3.08 × 10^−2^	1.53	POS
(E)-1,7-Bis(4-Hydroxyphenyl) Hept-1-En-3-One	0.48	3.14 × 10^−2^	1.48	POS
Thymidine-5′-Diphosphate	0.48	4.49 × 10^−2^	1.49	POS
Dihydrosphingosine	0.45	4.17 × 10^−2^	1.55	POS
STL519416	0.36	4.19 × 10^−2^	1.45	POS
PC (15:0/12-HEPE)	3.50	0.13 × 10^−2^	2.36	NEG
1,2-Dihydroxyheptadec-16-En-4-Yl Acetate	2.43	3.77 × 10^−2^	2.13	NEG
Deoxyuridine	2.41	4.56 × 10^−2^	1.61	NEG
Phosphatidylserine 18	2.23	2.52 × 10^−2^	2.06	NEG
Phosphatidylcholine 17	2.00	2.61 × 10^−2^	2.00	NEG
PC (17:1/13-HODE)	1.99	4.76 × 10^−2^	1.77	NEG
7,8-Dihydrobiopterin	1.61	4.52 × 10^−2^	1.95	NEG
(R, R)-Tartaric Acid	0.29	4.62 × 10^−2^	1.57	NEG

## Data Availability

Data are contained within the article and the Supplementary Materials.

## Supplementary Materials

The following supporting information can be downloaded at: https://www.mdpi.com/article/10.3390/ani14243672/s1. Figure S1: Proteomic features analysis in boar X/Y sperm; Figure S2: Peptide segment information in protein mass spectrometry data; Figure S3: Total ion chromatography(TIC) of QC sample; Figure S4: PCA validation and mass spectrometry results of metabolomics of boar X/Y sperm; Figure S5: Differential metabolites in amino metabolism of X/Y sperm; Table S1: Up-regulated differential protein-related metabolism in X/Y sperm; Table S2: Down-regulated differential protein-related metabolism in X/Y sperm.
