# Supplementary material for: Proteomic and Metabolomic Profiling Reveals Alterations in Boar X and Y Sperm
Source: Animals (Basel). 2024 Dec 19;14(24):3672. doi: 10.3390/ani14243672 (PMC11727386; doi:10.3390/ani14243672)
Supplement: Supplementary file 1 [file animals-14-03672-s001.zip › Supplement Tables.pdf]

**Supplement Tables**

**Table S1 Up-regulated differential proteins related metabolism in X-/Y-sperm**

| Protein symbol | KEGG pathway |
|---|---|
| FUT8 | Glycosaminoglycan biosynthesis keratan sulfate<br>N-Glycan biosynthesis |
| HSD17B4 | Primary bile acid biosynthesis |
| MTHFD2L | One carbon pool by folate |
| GGTA1 | Glycosphingolipid biosynthesis - lacto and neolacto series |
| FTL | Porphyrin and chlorophyll metabolism |
| ALDH18A1 | Arginine and proline metabolism |
| GSTK1 | Glutathione metabolism<br>Metabolism of xenobiotics by cytochrome P450<br>P450Drug metabolism - cytochrome P450 |
| PGK1 | Glycolysis / Gluconeogenesis |
| PEMT | Glycerophospholipid metabolism |
| COX7C | Oxidative phosphorylation |

**Table S2 Down-regulated differential proteins related metabolism in X-/Y-sperm**

| Protein symbol | KEGG pathway |
|---|---|
| COX1 | Oxidative phosphorylation |
| COX6A1 | Oxidative phosphorylation |
| ND2 | Oxidative phosphorylation |
| ATP4A | Oxidative phosphorylation |
| ATP5F1A | Oxidative phosphorylation |
| LOC100524239 | Oxidative phosphorylation |
| SDHC | Oxidative phosphorylation / TCA cycle |
| ND1 | Oxidative phosphorylation |
| CYTB | Oxidative phosphorylation |
| ST3GAL1 | Glycosaminoglycan biosynthesis keratan sulfate<br>Glycosphingolipid biosynthesis - globo series<br>Mucin type O-Glycan biosynthesis |
| MAN2B2 | Other glycan degradation |
| NT5E | Nicotinate and nicotinamide metabolism /<br>Pyrimidine metabolism / Purine metabolism |
| FTH1 | Porphyrin and chlorophyll metabolism |
| TKFC | Glycerolipid metabolism |
| ANPEP | Glutathione metabolism |
