# Supplementary figures and images for: Proteomic and Metabolomic Profiling Reveals Alterations in Boar X and Y Sperm
Source: Animals (Basel). 2024 Dec 19;14(24):3672. doi: 10.3390/ani14243672 (PMC11727386; doi:10.3390/ani14243672)

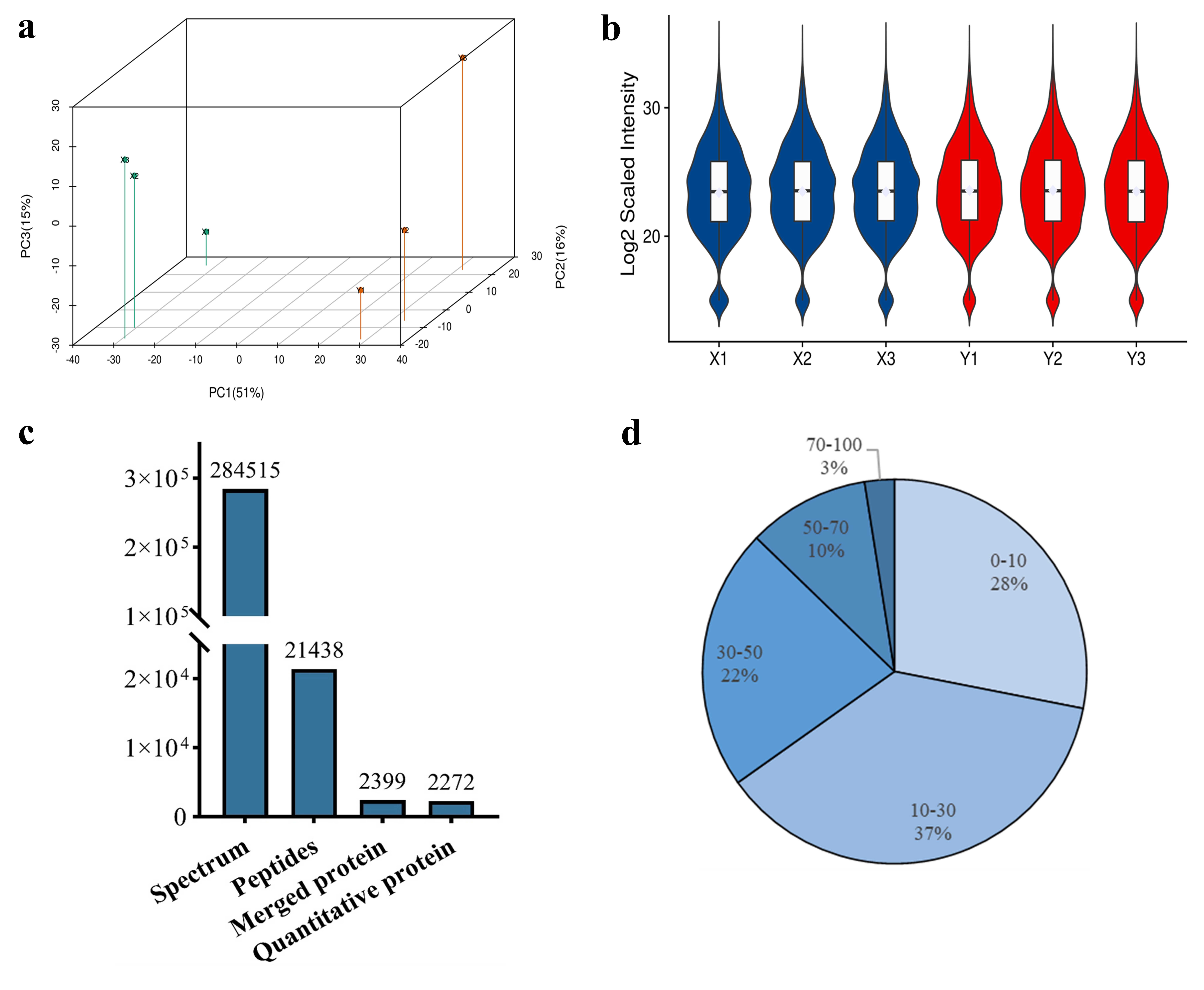

Supplement: Supplementary file 1 [file animals-14-03672-s001.zip › Figure S1.png]

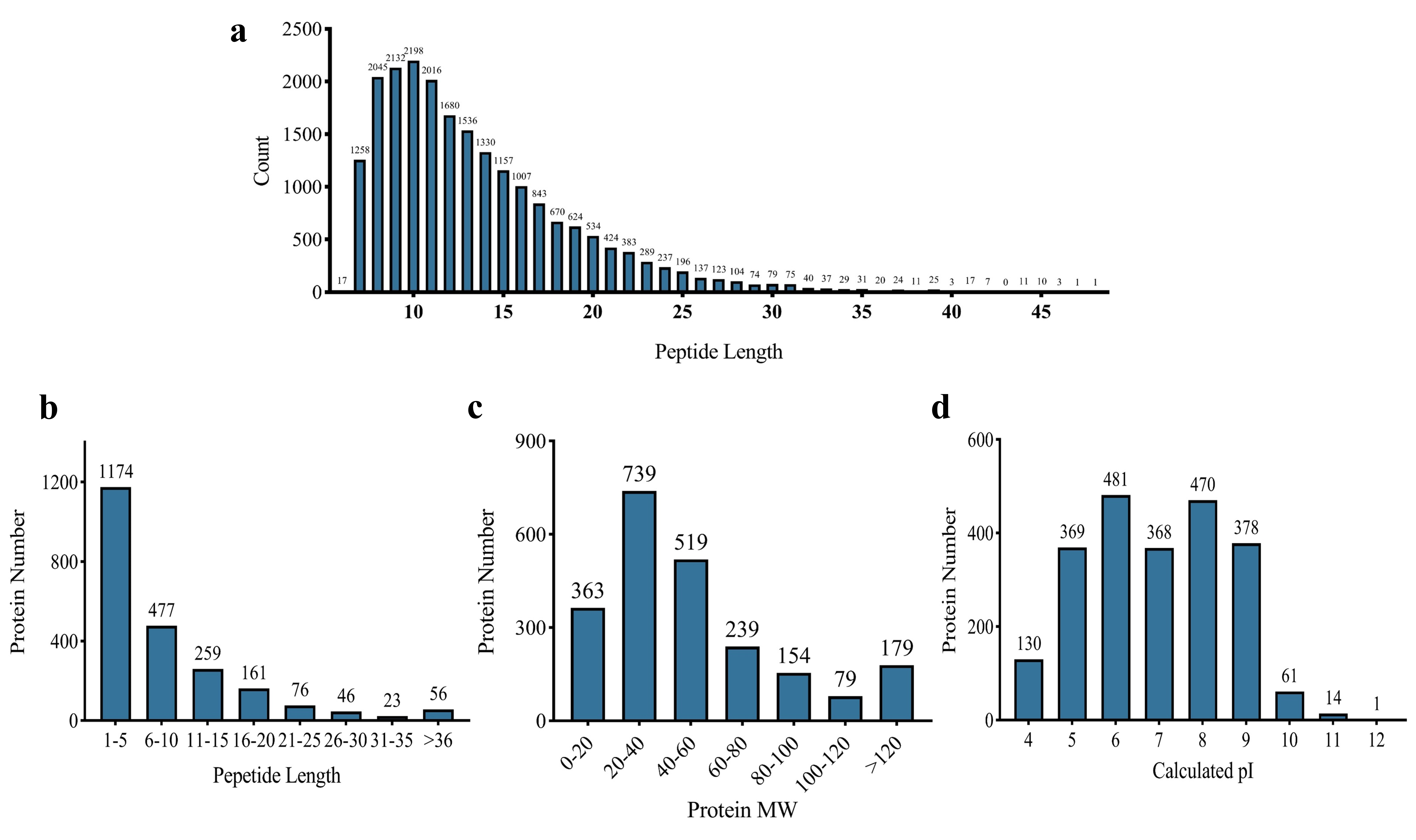

Supplement: Supplementary file 1 [file animals-14-03672-s001.zip › Figure S2.png]

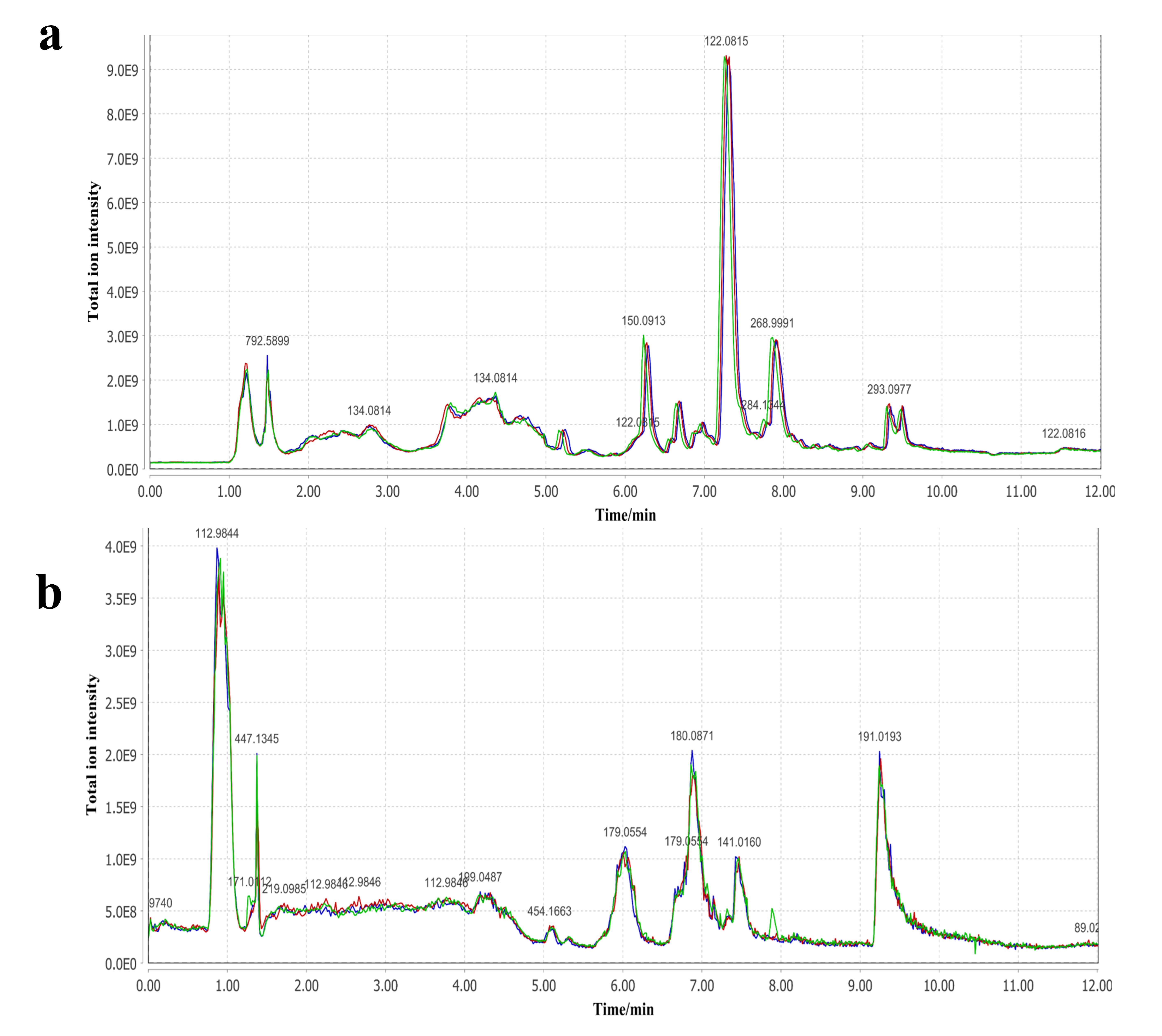

Supplement: Supplementary file 1 [file animals-14-03672-s001.zip › Figure S3.png]

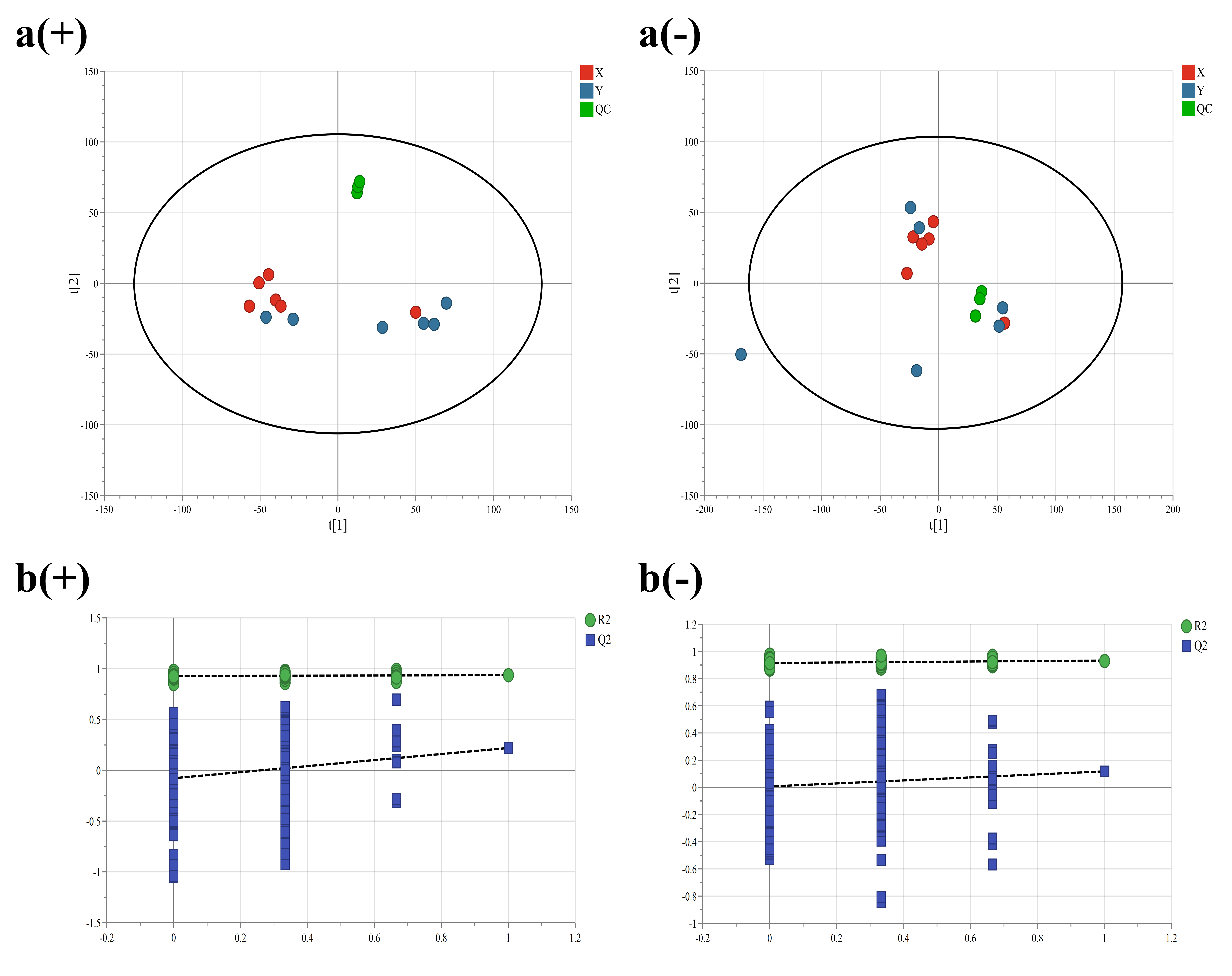

Supplement: Supplementary file 1 [file animals-14-03672-s001.zip › Figure S4.png]

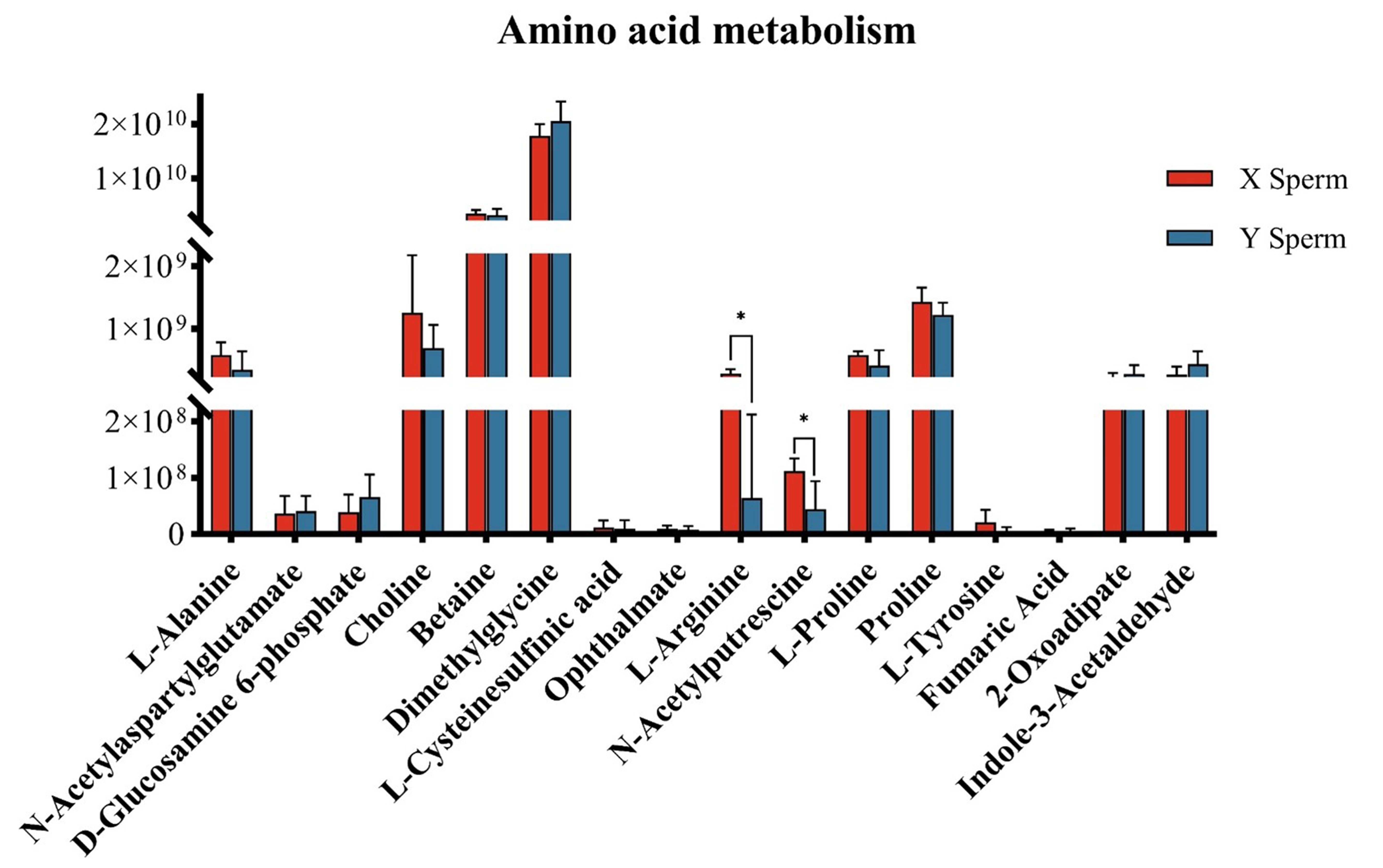

Supplement: Supplementary file 1 [file animals-14-03672-s001.zip › Figure S5.png]
